# Barriers and facilitators to the implementation of sit-less and move-more interventions within Australian primary healthcare settings

**DOI:** 10.1093/tbm/ibag025

**Published:** 2026-04-27

**Authors:** Aaron Beecroft, David Dunstan, Shannon Sahlqvist, Devdini Mohotti, Anna Chapman

**Affiliations:** Institute for Physical Activity and Nutrition, Deakin University, Geelong, VIC, Australia; Institute for Physical Activity and Nutrition, Deakin University, Geelong, VIC, Australia; Baker-Deakin Department of Lifestyle and Diabetes, Baker Heart and Diabetes Institute, Melbourne, VIC, Australia; Institute for Physical Activity and Nutrition, Deakin University, Geelong, VIC, Australia; Centre for Quality & Patient Safety Research, Institute of Health Transformation, Deakin University, Geelong, VIC, Australia; Centre for Quality & Patient Safety Research, Institute of Health Transformation, Deakin University, Geelong, VIC, Australia

**Keywords:** exercise, sedentary behavior, primary healthcare, general practitioners, nurses, implementation science

## Abstract

**Background:**

Due to the excessive burden and health costs of noncommunicable diseases, public health and clinical practice guidelines emphasize the importance of targeting population changes in risk factors of physical activity and sedentary behavior. Primary healthcare presents a pragmatic setting for embedding interventions targeting sitting less and moving more. However, to design effective implementation strategies for such interventions, it is necessary to understand the determinants that drive implementation in this setting.

**Purpose:**

To determine the barriers and facilitators influencing the implementation of sit-less and move-more interventions within Australian primary healthcare, as perceived by general practitioners and general practice nurses.

**Methods:**

Ten general practitioners and 10 general practice nurses completed online semi-structured individual interviews. Interviews were digitally transcribed and analyzed deductively; both the interview guide and analysis were guided by the Theoretical Domains Framework.

**Results:**

All domains within the Theoretical Domains Framework were represented. Notable barrier domains were: Environmental Context and Resources, related to reported time constraints; Skills, relating to limited formal training; Social Influences, relating to patient engagement challenges. Notable facilitator domains were: Beliefs about Consequences, reflecting perceived health benefits for patients with healthier movement behaviors, Environmental Context and Resources, including use of educational resources and integration of sit-less and move-more assessments within clinic workflows; and Social/Professional Role and Identity, highlighting how both general practitioners and general practice nurses have a role in implementation.

**Conclusions:**

These findings provide a foundation for future research focusing on the co-design of strategies supporting the implementation of such interventions within primary healthcare.

## Teaser

Strategies supporting the successful implementation of sit-less and move-more interventions within primary healthcare need to consider the time constraints, competing priorities, and lack of training that exist for clinicians. The development of practical resources, including posters and patient handouts, and the integration of an efficient screening tool within practice workflows, may help to facilitate implementation.

## Graphical abstract

**Graphical Abstract:**
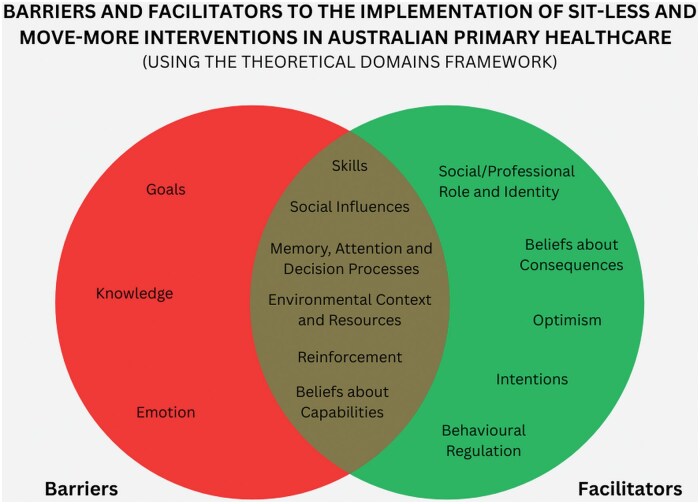

Implications

- **Practice:** Educational resources and integrated tools should be developed to support clinicians in efficiently delivering sit-less and move-more (SLAMM) assessment and advice within routine care.
- **Policy:** Policy initiatives should enable system-level support, such as funding appropriate training materials and embedding SLAMM screening tools in practice software to promote sustainable implementation.
- **Research:** Future studies should co-design and evaluate theory-informed implementation strategies that address the training, resource, and workflow challenges identified for SLAMM interventions in primary healthcare.

## Introduction

The health risks associated with insufficient physical activity and excessive levels of sedentary behavior are universally accepted [1, 2]. Meta-analytic evidence shows that high sitting time and low physical activity levels increase premature mortality and noncommunicable disease risk [2]. As such, current World Health Organization guidelines for adults recommend participating in at least 150 min of moderate-intensity, or 75 min of vigorous-intensity physical activity a week; along with completing muscle-strengthening activities on at least 2 days each week and limiting sedentary time [3]. Economic analysis indicates that insufficient physical activity participation and high amounts of daily sedentary behavior (>6 h/day) are associated with up to $850 million in healthcare costs annually in high-income countries [4, 5]. As a result, interventions targeting “sitting-less and moving-more” (also known by the acronym SLAMM) are needed.

Evidence-based SLAMM interventions have been successfully implemented in workplace settings [6, 7]. For example, lay member and office worker involvement was used to design and implement a successful office-based intervention involving height-adjustable desks, educational seminars and leaflets, and a booklet for action planning and goal setting to encourage employees to reduce their daily sitting time and increase daily activity [7]. Due to the large proportion of the population visiting primary healthcare services annually, various policy documents support the role of primary healthcare in promoting healthy movement behaviors [8, 9]. General practitioners (GPs) and general practice nurses (GPNs) make up most of the primary healthcare workforce, and both are trusted by patients to provide meaningful health advice [8, 10]. A recent review of reviews reported a small to medium positive effect for the effectiveness of physical activity promotion interventions, which were commonly delivered by GPs or other primary healthcare professionals [11]. Despite growing recognition of the health risks associated with sedentary behavior, few primary healthcare interventions have directly addressed this risk factor, with many solely focused on increasing physical activity [12, 13].

Many barriers to effective physical activity counselling within primary healthcare have been identified and need to be considered to support the implementation of sustainable SLAMM interventions. GPs and GPNs frequently cite lack of time, insufficient knowledge, skills and training, limited resources, and patients’ own resistance to behavior change as key obstacles [14–16]. Effective translation of evidence-based interventions into practice requires a nuanced understanding of context-specific barriers and facilitators that influence implementation. These insights can then be converted into practical, theory-informed implementation strategies. Because each primary care service operates within distinct systems, workflows, and resource constraints, implementation strategies must be tailored to local contexts to support the successful adoption and sustained implementation of interventions. These strategies represent the “how-to” of embedding interventions into routine care and should be selected through systematic approaches that link identified determinants to targeted actionable solutions.

Several frameworks exist to support this process, with the Theoretical Domains Framework (TDF) among the most frequently applied. Developed by integrating 33 theories related to behavioral change, the TDF provides a single, practical framework comprising 14 domains that capture key influences on behavior. The framework has been widely used to identify barriers and facilitators to implementation and inform the design of targeted implementation strategies to support the implementation of healthcare interventions [17–19]. While barriers and enablers to physical activity promotion in primary healthcare are relatively well documented, much less is known about what influences the implementation of SLAMM interventions. To address this gap, we applied the TDF to collect qualitative data from a sample of GPs and GPNs, aiming to identify the barriers and facilitators to the implementation of SLAMM interventions in Australian primary healthcare settings.

## Methods

### Study design and ethical approval

A qualitative study using individual semi-structured interviews was conducted. Ethical approval for the study was granted by the Deakin University Human Ethics Advisory Group-Health (HEAG-H 98_2023). The reporting of this study followed the Consolidated criteria for Reporting Qualitative research checklist [20].

### Participants and recruitment

A market research recruitment company (Focus People) was contracted to manage recruitment. Recruitment commenced on 16 September 2023 and closed on 24 October 2023. Recruitment was determined *a priori* and aimed to include 10 GPs and 10 GPNs across a range of Australian states and territories, age groups, and genders. Eligible participants were GPs and GPNs employed full-time or part-time in an Australian general practice clinic, with appropriate registration under the Australian Health Practitioner Regulation Agency, and an adequate understanding of the English language. The recruitment company used purposive sampling from its existing database to identify and screen eligible registrants. Participant information and consent forms were then distributed to those who met the inclusion criteria and expressed interest in participating. Those interested returned the completed consent form to the research team. Upon receipt of written consent, participants were emailed a unique Zoom link by the recruitment company to join their scheduled interview. If a participant missed their interview, the recruitment company re-contacted them to reschedule. Due to the limited details provided by the recruitment company, detailed screening flow data are unable to be provided. If a participant did not complete the interview, the company sought consent from another eligible registrant. Participants were offered financial compensation for participation in line with industry recommendations [21].

### Data collection

**Figure 1:**
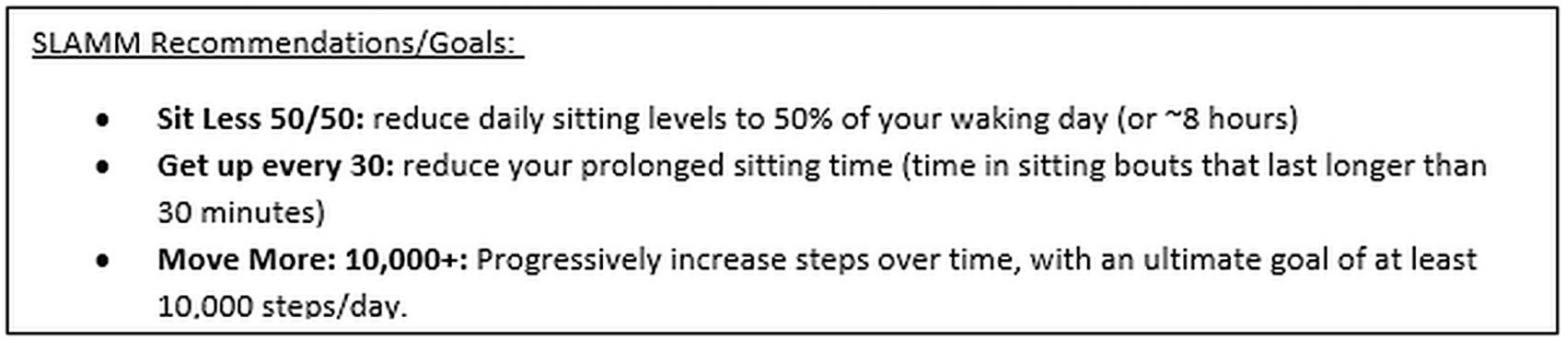
The sit-less and move-more recommendations provided to participants at the beginning of each interview.

Data were collected through individual semi-structured interviews conducted online via Zoom. The interview guide was developed in accordance with the five phases described by Kallio *et al*. [22]. At the start of each interview, the interviewer explained the importance of assessing patients’ movement behaviors (i.e. asking and recording individual physical activity and sedentary behavior levels), restated the project aims, and, if unfamiliar, outlined current SLAMM recommendations (Fig. 1). The interview schedule (Supplementary Appendix A) was structured to reflect the 14 domains of the TDF. Example questions for each TDF domain are provided in Table 1. Interviews were conducted by D.M. [a female Research Assistant (Bachelor of Biomedicine, Honours) with prior experience conducting qualitative interviews with GPs] and A.C. [a female Research Fellow (PhD) with expertise in implementation science and prior experience applying the TDF in qualitative interviews with GPs and GPNs]. Interviewers had no prior relationships with study participants. All interviews were audio recorded, de-identified, and digitally transcribed by a professional transcription service. Due to the reported misalignment with reflexive thematic analysis, data saturation was not discussed [23]. The authors focused on ensuring sufficient depth and richness of data to enable robust and meaningful analysis. Transcripts were not returned to participants because consent covered participation up to the conclusion of the interview and did not include post-interview transcript review.

**Table 1:** Example interview questions for each TDF domain used as part of the semi-structured interview schedule.

TDF domain	Example interview question
1. Knowledge	Of the key SLAMM recommendations could you please tell me which you are/are not knowledgeable about?
2. Skills	Have you had training regarding how to assess and manage patients with regard to sedentary behavior (sitting time) and physical inactivity?
3. Social/Professional Role and Identity	What is your role in the assessment of sedentary behavior (sitting time) and physical activity? And the role of others in your clinic?
4. Beliefs about Capabilities	How confident are you that you can assess patients for sedentary behavior/physical inactivity and then provide tailored SLAMM advice?
5. Optimism	How optimistic are you that assessing and providing tailored advice according to the SLAMM recommendations would improve outcomes for patients?
6. Beliefs about Consequences	What do you think the benefits are for assessing sedentary behavior/physical inactivity and then providing tailored advice to patients, for… You? Patients? Your Clinic?
7. Reinforcement	Are there any incentives/rewards that influence the proactive assessment of sedentary behavior/physical inactivity?
8. Intentions	In the next month, how strong is your intention to proactively assess and provide tailored SLAMM advice?
9. Goals	When setting goals around your performance at work, how much does the assessment and provision of care related to physical inactivity/sedentary behavior feature in such goals (prompt for short/long term)?
10. Memory, Attention, and Decision Processes	How much attention do/would you have to pay to proactively assess or provide tailored SLAMM advice?
11. Environmental Context and Resources	Are there any aspects of your clinic environment that affect your ability to assess people’s level of risk for sedentary behavior/physical inactivity [with risk screening tools]?
12. Social Influences	You work alongside other GPs, practice nurses, allied health staff etc.—How do they influence whether or not you proactively address patients sedentary/physical activity behaviors [prompt for assess/provide tailored SLAMM interventions]?
13. Emotion	Does the thought of not addressing sedentary behavior/physical inactivity create any feelings of worry or concern in you?
14. Behavioral Regulation	Are there any procedures/steps or ways of working that would encourage you and your colleagues to proactively assess and provide tailored SLAMM advice?

### Data analysis

The interview transcripts were systematically analyzed using QSR NVivo, via a deductive approach aligned with all 14 domains of the TDF (Table 1). A.B. and D.M. independently coded each transcript to identify content relevant to the 14 TDF domains. Within each domain, A.B. derived themes from the initial coding and then classified them as either a barrier or a facilitator. Barriers were defined as personal or systemic factors that were perceived to prevent or impede participation in, or implementation of, interventions [24]. Facilitators were personal or systemic factors that were perceived to assist or allow for participation in, or implementation of, interventions [24]. The identified barrier or facilitator themes were presented alongside representative quotes to ensure the authenticity and credibility of the data. All analytical steps involved ongoing review between A.B., D.M., and senior author A.C. to further enhance analytical credibility. Participants were not invited to provide feedback on the thematic analysis results.

## Results

### Participant characteristics

The sample included 20 participants: 10 GPs and 10 GPNs across a range of Australian states and territories (Table 2). Interviews averaged 38 min, ranging from 27 to 59 min. GPs’ mean age was 41 years, ranging between 27 and 64 years; while GPNs’ mean age was 43 years, ranging between 26 and 55 years. Most participants were female (18/20), with the two male participants both GPs. Employment status varied; nine GPs and only three GPNs were employed full-time. The GPs had an average of 13 years’ experience, ranging from 0.7 to 35 years; similarly, the GPNs had an average of 12 years’ experience, ranging from 2 to 33 years.

**Table 2:** Participant characteristics.

Participant	Age	Gender	Location (state)	Employment status	Years of experience
GP	63	F	VIC	Full-time	30
GP	29	F	NSW	Full-time	0.7
GP	64	M	NSW	Full-time	35
GP	27	M	NSW	Full-time	2
GP	35	F	NSW	Part-time	5
GP	54	F	NSW	Full-time	26
GP	27	F	VIC	Full-time	2
GP	61	F	VIC	Full-time	27
GP	27	F	NSW	Full-time	2
GP	27	F	NT	Full-time	1.5
GPN	53	F	VIC	Full-time	30
GPN	51	F	VIC	Part-time	13
GPN	52	F	VIC	Part-time	10
GPN	42	F	QLD	Full-time	4
GPN	31	F	SA	Part-time	6
GPN	42	F	VIC	Full-time	7
GPN	47	F	VIC	Part-time	6
GPN	55	F	VIC	Part-time	33
GPN	34	F	NSW	Part-time	5
GPN	26	F	WA	Part-time	2

### Barriers and facilitators

Aligned with the TDF, the following section presents the barriers and facilitators to the implementation of SLAMM interventions within general practice. Table 3 summarizes the TDF domains that were identified as barriers and/or facilitators, along with brief descriptors. The most relevant domains are described in greater detail below:

**Table 3:** Identified barriers and facilitators to the implementation of SLAMM interventions, as mapped to the TDF.

Domains	Identified barriers	Identified facilitators
1. Knowledge	Only familiar with general concepts	
2. Skills	No formal or specific training Less skilled in sedentary behavior advice	Can provide general physical activity advice
3. Social/Professional Role and Identity		GPs and GPNs both have a role Can refer to allied health professionals for further support
4. Beliefs about Capabilities	Less confident in providing tailored advice	Confident in assessing/screening
5. Optimism		Optimistic about potential outcomes
6. Beliefs about Consequences		Benefits to health outcomes Clinician satisfaction Benefits to the clinic and healthcare system
7. Reinforcement	No current incentive for intervention	Use of current government-subsidized health assessments
8. Intentions		Intention to implement once knowledgeable
9. Goals	Competing priorities	
10. Memory, Attention, and Decision Processes	Requires conscious effort	Concepts are easy to remember Prompts on when to implement
11. Environmental Context and Resources	Time constraints Room availability/capacity	Waiting room posters Resources and handouts Clinical software integration Use of government-subsidized health assessments
12. Social Influences	Patient engagement	Workplace support
13. Emotion	Stress affects implementation	
14. Behavioral Regulation		Reminders and follow-up appointments

#### Social/professional role and identity

GPs and GPNs reported that the provision of SLAMM assessment and advice was an appropriate part of their role.

*I feel I do have a role. Often with the new patients, they will see one of our practice nurses first, and with a general overview, one of the questions as part of our template is “how much exercise do you do?” But I think this is a question that’s still applicable to patients that aren’t new as well, and I definitely have a role in discussing that with all patients that come through.* (GP08)

Participants from both groups suggested that the implementation of a SLAMM assessment and advice by GPNs was best facilitated when completing specific government subsidized services for patients, including Health Assessments (which include assessments for those 40–49 years and at risk of type 2 diabetes; 45–49 years and at risk of chronic disease; or those 75 years and older); and GP chronic condition management plans (which allow patient’s with one or more chronic conditions five subsided visits per calendar year to an allied health professional to assist with managing their condition) [25–28].

*I think, too, it would be good to approach practice nurses as well, because, as I said, they’re often the first person that the patient sees, particularly when it comes to these health assessments for older people and GP management plans for people with chronic illnesses.* (GP09)

Despite the overall acknowledgment that the provision of SLAMM assessment and advice was appropriate for their role, some GPNs suggested that they should play a leading role in this area, while other GPNs felt that GPs have the primary and most influential role.

*I think it’s really more the role of the practice nurse kind of to hone in on those lifestyle behaviours…. Like, we work together, don’t get me wrong, we work really closely together. But I just feel like it’s an opportunity for the practice nurse to kind of hone in on those things that the GP is just not going to have time to do.* (GPN09)

*When we perform these assessments and make recommendations, the patient always goes back to their GP and discusses them. So we make recommendations, then the GP moves forward and discusses it further, makes referrals and things like that. So I suppose we are in that role, but it’s the GP that finalises everything always.* (GPN08)

Both GPs and GPNs spoke about how referrals to allied health professionals, such as exercise physiologists, assist with the provision of physical activity advice and subsequent management.

*In my practice, I do often refer on to an exercise physiologist or to a physiotherapist to do the appropriate exercise plan. I probably do sort of shift that responsibility on to them a little bit.* (GP14)

#### Knowledge

Despite broad agreement that the provision of SLAMM interventions is appropriate in their role, GPs and GPNs reported only having basic knowledge and awareness regarding the concepts of SLAMM assessments and advice, stating that they needed further guidance on specific physical activity and sedentary behavior recommendations. For example, when asked if they had any prior knowledge of SLAMM recommendations, one GPN said:

*I’ve heard about them generally, but if you’d asked me beforehand [….] I kind of would have gone, “Oh, I’m not quite sure exactly what the recommendations are.”* (GPN12)

#### Skills

GPs and GPNs reported having skills in providing general physical activity guidance to their patients, including tailoring advice to the patient’s current ability and the need for graded increases in physical activity completion.

*I say to patients that they should try and sort of exercise or walk for an hour most days of the week, so four to five days of the week. It’s the general guidelines that I have. But again, I do tailor that to people’s needs…… I might say to them, "Look, start by just walking up and down the street or around the block for 5 or 10 minutes a day with a view to gradually increasing that and increasing your pace and things like that."* (GP06)

However, both professions reported being less skilled in assessing and providing specific advice regarding sedentary behavior.

*The focus would be on what exercise they do, and that will include things like housework, gardening, but there’s never a question that is directly how much you’re sitting. That’s something that’s definitely missing.* (GPN06)

Neither profession stated that they had received any formal or specific training regarding assessing or providing SLAMM advice. Some participants acknowledged a need to receive further training on sedentary behavior. For example, when asked if they would like further training on the SLAMM recommendations, one GPN reported:

*I would think so. Especially when you’re talking about the assessing for sedentary behaviour and things like that.* (GPN15)

#### Beliefs about capabilities

Despite acknowledging the need for further upskilling, most GPs and GPNs voiced confidence in their ability to conduct a patient assessment or screening for sedentary behavior or insufficient physical activity.

*I’m pretty confident because I’m confident to ask the question, because like I said, it’s just a question.* (GPN13)

That being said, some GPs and GPNs reported being less capable of providing individualized programs to patients as part of a SLAMM intervention, often mentioning a need for further training or resources to assist.

*I think I feel reasonably comfortable with assessing, but at the moment I think I would like some more resources or training with regards to providing a personalised program.* (GP08)

#### Beliefs about consequences

GPs and GPNs conveyed that the implementation of a SLAMM intervention would greatly improve health outcomes for patients by helping to promote independence, prevent illness, and prevent or manage common chronic diseases.

*…the benefits to the patient are obviously health benefits in every aspect. Sleep, mental health, cardiovascular health, bone health, everything.* (GP11)

*The benefits to the patient, particularly in that older cohort, is staying at home. I mean, that’s pretty huge, and that—I think for a lot of them, that’s what eventually forces them into care in that they can no longer—whether it’s get up off the floor, cook for themselves, even with supports in the house, if they can no longer manage, so for that older age group, it’s crucial that they’re active.* (GPN06)

Some GPs and GPNs voiced that the use of a SLAMM intervention and its consequential benefits for their patients would make them feel good about providing that level of care.

*….particularly when you can see some changes in people’s behaviour, it makes me feel good. It’s nice always to get some positive feedback in your professional life. So it does make me feel better. It makes me feel good.* (GP09)

Beyond benefits to the individual, GPs reported that incorporating SLAMM interventions within their consultations would create positive perceptions from patients and ‘lift the profile of the practice’ (GP09) due to providing ‘good quality health care’ (GP08). GPNs also noted that implementation would benefit the entire primary healthcare system by reducing the need for future appointments via improved movement behaviors and related health outcomes, achieving overall cost savings.

*If I’m looking at it from a comprehensive whole Australian health care, with that hat on, it’s definitely a positive, it really is. The less admissions we have in this country, the better our healthcare system is because it’s already at the point where it’s a little bit broken. So if we can try and minimalise some of the unnecessary visits, then that’s just a positive really.* (GPN11)

#### Social influences

Both professions discussed how patients’ own engagement with SLAMM recommendations was a barrier to successful implementation, noting that patients may not recognize the importance of making changes.

*But again, it’s a matter of whether they’re going to listen to my advice and whether they’re going to implement it.* (GP09)

*I think it would probably come down to the patient’s level of motivation to want to make change, to even see that there’s an issue and to want to engage with any of your recommendations…. I feel confident in being able to identify and make recommendations and have a discussion, but I think it does come down to the patient’s level of motivation and will to change. They’re your [the clinician’s] barriers.* (GPN10)

However, GPNs reported that providing coordinated advice alongside GP colleagues would assist with the implementation of SLAMM interventions, noting teamwork as a facilitator.

*I think the general practitioners, they would mention it to the patient, and the patient– they are quite open to seeing the GP, and they already had a rapport with the doctor…, then from there they group in the nurses. I think things like that, working as a team, really helps as well.* (GPN07)

#### Goals

Both professions noted that competing demands, which depended on the presenting patient’s concerns, impacted their prioritization of SLAMM advice.

*I think it comes under lifestyle for me. And that’s always a very high goal, because I don’t necessarily believe in having medication for everything. I’d rather prevent the problems.* (GP11)

*I think that I’d say probably medium because there are just so many competing interests.* (GPN12)

#### Environmental context and resources

Being ‘time-limited with patients’ (GPN09) and the competing priorities in primary healthcare were key barriers. GPs, in particular, expressed concern about having sufficient time to provide SLAMM assessments and advice during routine consultations, especially when patients were attending for other reasons.

*Look, time is always an issue in general practice and patients might come in for a few different issues and that’s not on their agenda to address, and then you feel that that’s something that’s important.* (GP14)

In addition to limited time, GPNs highlighted the availability and capacity of clinic rooms as a potential barrier, given the need for private, individual conversations with patients.

*I think the big factor is time and availability, because what we have is one treatment room for us personally and there’s three nurses, but we can’t all be in there at the same time. So I might have somebody that I want to do [an] assessment on, but I can’t do it because the other nurse is doing a dressing or something like that. And so we have to sort of overcome that barrier in order to be able to run a program like the one you’re describing.* (GPN08)

GPNs discussed how the completion of the specific government subsidized assessments or plans (which they are commonly involved in) provided the best opportunity to facilitate an assessment and the provision of SLAMM advice to their patients.

*So when we’re doing health assessments, whether it’s with the elderly, whether it’s the cardiac, whether it’s the 40-to-45 or 45-to-49-year-olds, I can’t remember now, but there’s cohorts of groups that we assess on a regular basis. So we can always bring in that discussion point at that time.* (GPN08)

Further, both GPs and GPNs expressed support for displaying posters in clinic waiting rooms to promote SLAMM interventions, noting that such materials would help raise awareness among patients and staff, and provide a prompt for action.

*…some sort of poster for the waiting room would be useful because that prompts the patients. Because if I forget the patient would be prompted. And also, there are other doctors in the practice so it would prompt other patients of other doctors to ask them as well.* (GP09)

GPs and GPNs also reported that having access to SLAMM-specific handouts would facilitate delivery of SLAMM recommendations, and for GPs, a sense that this would also promote better SLAMM behaviors in their patients.

*You need to have some little leaflets that you maybe can hand out, that the doctor can hand out to the patient - something that they can take home. Because then, it’s always good if they have something in their hand that they can refer to when they’re at home. …Often when they leave the doctor’s door, they forget what was said.* (GP09)

Both professions were also positive about the use of prompts or tools embedded within clinical software to assist with screening and assessment.

*So I think the biggest one would be having the SLAMM assessment in Best Practice* [a common practice management software] *- would be a great step forward.* (GPN15)

#### Memory, attention, and decision processes

GPs and GPNs supported the use of screening tools and prompts to help keep SLAMM interventions front of mind, as both professions reported that delivering such interventions would require conscious effort. GPs also suggested that reminders [either during consultations or within the clinic (i.e. via posters)] or a specific protocol would facilitate this.

*At the moment, it’s not automatic, it would be intentional, but if it could be implemented well into the workflow that’s something I’d like to make fairly automatic.* (GP13)

GPNs did report that SLAMM concepts were easy to remember, which may help facilitate implementation. When asked if the SLAMM recommendations were easy or difficult to remember, one GPN said:

*I think they are all equally easy to remember. Yeah, they’re pretty straightforward, I would say.* (GPN07)

GPs spoke about the situations where they may be prompted or more likely to assess and provide SLAMM advice, including interactions focused on weight management and cardiovascular disease risk.

*I would generally recommend, particularly when it comes to cardiovascular risk screening, I will talk about exercise recommendations […….] That’s when it comes up most frequently…* (GP08)

#### Reinforcement

When discussing incentives to support implementation, both GPs and GPNs spoke about how aspects of the intervention could be implemented as part of the current government-subsidized health assessments. GPNs did note that currently, these incentives apply only to the GP or general practice clinic, and not to the GPN themselves.

*We do the GP management plans. We do the health assessments - there are also the health assessments that we do for people at risk of diabetes and the 45-to-49-year-old health assessments, the risk for heart disease. So we get a financial incentive when we when we do that. Looking at exercise, moving more, that’s all part of that assessment.* (GP09)

*It probably would because you’ve got to remember though, that the incentives go to the doctors, not the nurses who do it. So when you say that incentive, it’s no incentive to me, but it would be incentive to the doctors, and then the doctors would be the ones that would probably instigate this, or want this to happen.* (GPN11)

GPs addressed that there is no specific financial incentive supporting the delivery of SLAMM interventions, adding that a specific government subsidized service would be a facilitator.

*If they can have an item number to specifically encourage this, then that would make the access barrier a lot less.* (GP15)

GPNs also supported that monetary-based incentives that benefit either the practice and/or patient may help facilitate a SLAMM intervention.

*How much does it cost to the patient? Are we making this a [government] rebated assessment? Because sometimes some people would be more open to a free health check– and then you can incorporate that into it, but if they’re having to pay, they might be less likely to want to engage in it.* (GPN10)

## Discussion

This research project aimed to determine the barriers and facilitators influencing the implementation of SLAMM interventions within primary healthcare using the TDF. Data from the individual interviews were mapped to all 14 domains of the TDF. These findings provide a foundation that could assist with the identification of strategies to support the successful implementation of SLAMM interventions. The notable domains were identified as those most consistently reflected in participants’ accounts as influencing the successful implementation of SLAMM interventions in primary healthcare. The three notable barrier domains were Environmental Context and Resources (Domain 11), Skills (Domain 2), and Social Influences (Domain 12). The three notable facilitator domains were Beliefs about Consequences (Domain 6), Environmental Context and Resources (Domain 11), and Social/Professional Role and Identity (Domain 3).

### Barrier domains

Time constraints (Environmental Context and Resources) were the most commonly reported barrier. This was closely related to the many competing priorities (Goals) that exist for both GPs and GPNs within primary healthcare. This was despite clinicians reporting that having discussions with patients about SLAMM was a high priority for them. Time constraints for both GPs and GPNs are frequently reported in the literature [14, 16]. A narrative review on the barriers and facilitators of physical activity promotion in primary healthcare reported lack of time as a major barrier for English GPs, with similar findings also reported among Australian GPs [14, 15]. Similarly, a qualitative study involving 13 UK nurses exploring their perceptions on providing physical activity advice reported that time pressures were an extrinsic barrier [16]. Overall, these findings highlight the need for strategies assisting the implementation of SLAMM interventions to be time sensitive and allow for efficient integration alongside the many priorities that exist within primary healthcare. While GPs and GPNs voiced their confidence in being able to assess physical activity and sedentary behavior levels among patients (Beliefs about Capabilities), both professions expressed a need to be upskilled in providing advice to patients based on this assessment to promote changes in their movement behaviors, especially concerning sedentary behavior change (Skills). Our findings support those of previous studies highlighting that a lack of formal training in physical activity persists for both GPs and GPNs, while adding that specific training on screening and advising sedentary behavior change is also required in order to support the implementation of SLAMM interventions within primary healthcare [16, 29]. The upskilling of clinicians may also help address the identified patient engagement barriers reported (Social Influences). Patient’s motivation for, and resistance to, behavior change have been previously identified as perceived barriers to the provision of physical activity counselling by both GPs and GPNs [15, 30]. However, tailoring SLAMM advice to patients’ individual circumstances and the use of a “piecemeal” approach to physical activity provision, as discussed by UK nurses in optimizing the delivery of physical activity advice, may be considered as necessary skills for clinicians to help address these barriers [16].

### Facilitator domains

The notion that both GPs and GPNs have a role in implementing a SLAMM intervention within primary healthcare was found to be a facilitator (Social/Professional Role and Identity). While it is well acknowledged by GPs that the provision of physical activity advice to patients is a necessary part of their role within primary healthcare, the role of GPNs in assessing and providing SLAMM advice is less understood [15, 28]. Some previous research has explored the role and efficacy of utilizing GPNs in delivering sedentary behavior and physical activity interventions, with process evaluation findings indicating GPNs valued their involvement within the interventions, which included receiving training on facilitating behavior change for patients [31, 32]. Our findings reinforce this and suggest that both GPs and GPNs believe that GPN’s role can include physical activity and sedentary behavior assessment and advice for patients; however, further research may be required to explore their role in the implementation of SLAMM interventions.

The findings also determined that clinicians understood the varying physical, social, and mental health benefits for their patients by promoting SLAMM (Beliefs about Consequences). The provision of resources, such as clinic posters promoting SLAMM and handouts for clinicians to distribute to patients, was seen as a facilitator for integrating SLAMM interventions (Environmental Context and Resources), acting as prompts for clinician–patient discussions (Memory, Attention, and Decision Processes). The provision of these resources was regarded as having a further benefit of keeping the SLAMM intervention “front of mind.”

Another proposed facilitator that emerged as a potential prompt for both GPs and GPNs was embedding an assessment of patients’ physical activity and sedentary behavior within the practice management software (Environmental Context and Resources). This development may allow for efficient screening of patients’ movement behaviors and provide a template for further discussion on behavior change if required. This SLAMM screening may also be incorporated (if not already) within the government-subsidized health assessment services that exist, as these appointments were reported as a current opportunity to facilitate discussion on SLAMM for patients. A physical activity screening tool for integration within electronic medical records has been previously developed by Clark *et al*. [33], using a Participatory Action Research design involving Canadian primary care professionals, patients, and researchers [33]. The usability testing of the screening instrument, which focused exclusively on physical activity and was centered around the Canadian physical activity guidelines, highlighted the importance of using simple questions within the instrument and the requirement for the instrument to integrate seamlessly within the existing workflow of primary healthcare [33, 34]. Although the effectiveness of this tool in supporting the provision of physical activity advice is unknown, an integrated screening tool (e.g. the Physical Activity and Sitting Time Balance Index—PASTBI) may be a potential strategy to help facilitate the implementation of SLAMM interventions within primary healthcare [35].

The identification of notable barrier and facilitator domains provides actionable direction for implementation planning. Strategies that address environmental constraints, such as embedding SLAMM screening within practice management software and aligning discussions with routine health assessments, may help mitigate time pressures. Targeted professional development focused on sedentary behavior counselling and brief behavior change techniques may address identified skill gaps, while practical prompts (e.g. posters and patient resources) may support clinician–patient engagement. These recommendations align with evidence from effective workplace SLAMM interventions, where multicomponent strategies combining environmental restructuring, prompts, and training have supported sustained behavior change [6, 7]. Translating these principles to primary healthcare settings may enhance the feasibility and sustainability of SLAMM implementation.

### Strengths and limitations

This study is strengthened by the use of the TDF framework as a template for the interview schedule and in guiding the investigation of the barriers and facilitators to the implementation of SLAMM interventions, which assists in the identification of theory-informed implementation strategies. The findings from this study also contribute important evidence regarding the implementation of interventions targeting sedentary behavior change, highlighting a need for further education for clinicians on the importance of sedentary behavior reduction. Participants were recruited from a majority of Australian states and territories, ensuring wide applicability. From the participant sample, only two identified as male, limiting the diversity of gendered perspectives, which may affect the transferability of findings. The predominance of female GPNs reflects the gender distribution of the broader Australian nursing workforce [36]. Findings may apply to primary healthcare systems in other high-income countries; however, generalizability may be limited for lower-income settings. Also limiting the study is that findings reflect participants’ perceptions rather than the actual determinants of implementation following the delivery of an intervention, which should be considered when interpreting the results.

## Conclusion

We utilized the TDF to identify the barriers and facilitators that are perceived by GPs and GPNs to influence the implementation of SLAMM interventions within Australian primary healthcare settings. The findings provide a foundation for future research to co-design strategies that may support the successful implementation of SLAMM interventions. As highlighted in this study, such strategies will need to consider the time constraints and competing demands that exist for GPs and GPNs working within primary healthcare, along with their lack of training in providing patients with advice relating to physical activity and sedentary behavior change. The use of practical resources such as posters and educational handouts, as well as the integration of a SLAMM assessment tool within practice management software, may assist in facilitating the sustainable implementation of such interventions.

## Supplementary Material

ibag025_Supplementary_Data

## Data Availability

Requests for access to data from the study can be made by contacting the corresponding author.

## Welfare of animals

This article does not contain any studies with animals performed by any of the authors.
