# Supplementary material for: Barriers and facilitators to the implementation of sit-less and move-more interventions within Australian primary healthcare settings
Source: Transl Behav Med. 2026 Apr 27;16(1):ibag025. doi: 10.1093/tbm/ibag025 (PMC13120876; doi:10.1093/tbm/ibag025)
Supplement: ibag025_Supplementary_Data [file ibag025_supplementary_data.docx]

Appendices

Appendix A: Schedule for Interview with Primary Care Providers

**Introduction**

Thank you for agreeing to participate in an interview.

May I confirm that you’ve read the Participant Information Sheet that was provided to you? {*if consent form not received, ask for them to submit consent form*}

Do you have any questions about the interview or the research project overall?

I’d like to remind you that you may cease the interview at any point and/or withdraw from the study at any time, without any repercussions. You may also decline to answer any questions you’d prefer not to answer.

I’ll be recording the interview for transcription purposes – are you happy to make a start?

(*Commence recording*)

For the recording, could you please confirm that you consent to participate in this interview?

**Preamble**

For some context for this interview, we know that high levels of sitting time as well as a lack of exercise are distinct risk factors for multiple chronic diseases. We also know that effective risk screening and personalised interventions are fundamental to improve these dual risk factors.

Sit Less and Move More (SLAMM) interventions have been developed and successfully implemented to address high levels of sitting and physical inactivity, but these interventions have not yet been tailored to the primary care setting. The overall aim of this project is to adapt an existing SLAMM intervention, and then co-design strategies that will assist primary care providers to address sedentary behaviour and physical inactivity within primary care.

This project is being conducted in different phases. In this phase, we are interested in learning from you (and we will also be asking other GPs and practice nurses), about what would make it easy or difficult to assess patients for physical inactivity/sedentary behaviour, and then implement personalised SLAMM interventions.

I’ll be asking a variety of questions today that are derived from different theories of human behaviour. These questions will assist us to determine which factors best apply to Sit Less and Move More in the primary care setting. The information that we will get from this phase will help us to co-design some strategies that will hopefully assist primary care providers to implement SLAMM interventions within primary care.

**Interview questions**

| Prior to this study, how familiar were you with Sit Less and Move More recommendations/goals? |
| --- |
| If familiar:   - Can you tell me what your general views are on the recommendations/goals? - How evidence-based do you think SLAMM recommendations are? |
| If low-level of familiarity: [explain SLAMM recommendations further to participant; provide detail on key recommendations or show slide]  SLAMM Recommendations/Goals:  Sit Less: **50/50:** reduce daily sitting levels to 50% of your waking day (or ~8 hours)  **Get up every 30:** reduce your prolonged sitting time (time in sitting bouts that last longer than 30 minutes)  **Move More:** **10,000+:** Progressively increase steps over time, with an ultimate goal of at least 10,000 steps per day. **Active hours:** take a 3-minute active break every hour via a short walk or a set of simple resistance activities.   - Do you use any other recommendations in your work regarding sedentary behaviour/physical inactivity? What are they? |
| *As previously mentioned, we would like to find out more about what you think makes it easy or difficult to assess patients and then provide care that is in line with these recommendations.* |
| **Knowledge (1)** |
| - Of the key SLAMM recommendations could you please tell me which you are/are not knowledgeable about [use prompt slide of key recommendations]? |
| - For the SLAMM recommendations that you are knowledgeable about, are there any gaps in what you know? |
| **Social/Professional Role and Identity (2)** |
| - What is your role in the assessment of sedentary behaviour (sitting time) and physical activity? And the role of others in your clinic? |
| - What is your role in the provision of personalised SLAMM interventions for patients? And the role of others in your clinic? |
| - To what extent is the assessment and subsequent management of sedentary behaviour (sitting time) and physical activity an appropriate part of your professional role?   - Would you consider the assessment component or intervention component any more/less appropriate to your role? |
| **Skills (3)** |
| - Have you had training regarding how to assess and manage patients with regard to sedentary behaviour (sitting time) and physical inactivity?  1. If yes, can you tell me about the training you have received to date  [prompt for content, facilitator, and frequency]? 2. Are there any specific areas you would you like to receive training (or additional training) in? (Prompt: sedentary behaviour/sitting time?) (Prompt: assessment or management?) |
| - Do you have the skills to:   - Use risk assessment tools to screen patients for sedentary behaviour (sitting time) and physical inactivity?   - Provide patients with tailored SLAMM advice?   - Refer to exercise professionals (where relevant)?  1. If not to any of the above, what skills would you require to achieve this? |
| **Beliefs about capabilities (4)** |
| - How confident are you that you can assess patients for sedentary behaviour/physical inactivity and then provide tailored SLAMM advice?  1. If confidence low for particular topic area: what would make you feel more confident? Is there anything that would increase your confidence? 2. If confidence high for particular topic area: how confident are you in maintaining or enhancing your existing practice? |
| - How difficult or easy do you feel it is to:  1. assess patients for patients for sedentary behaviour/physical inactivity? 2. provide tailored SLAMM advice to patients? |
| **Beliefs about consequences (5)** |
| - What do you think the benefits are for assessing sedentary behaviour/physical inactivity and then providing tailored advice to patients, for…  1. You? 2. Patients? 3. Your Clinic? |
| - What do you think the downsides are for assessing sedentary behaviour/physical inactivity and then providing tailored advice to patients, for …  1. You? 2. Patients? 3. Your Clinic? |
| - In your opinion, do the benefits of addressing sedentary behaviour/physical inactivity outweigh the downsides? |
| **Goals (6)** |
| - Considering other tasks and priorities that you have, how important is it for you to: (Prompt: Scale of 1 (not important) to 10 (extremely important), Why?)  1. Assess sedentary behaviour/physical inactivity of patients? 2. Provide comprehensive and personalised SLAMM advice to patients according to their risk? |
| - When setting goals around your performance at work, how much does the assessment and provision of care related to physical inactivity/sedentary behaviour feature in such goals (prompt for short/long term)? |
| **Memory, attention and decision processes (7)** |
| - How much attention do/would you have to pay to proactively assess or provide tailored SLAMM advice? [Prompt: automatic or requires intentional thought/decision] |
| - Are there any specific components of SLAMM recommendations that are more easy/difficult to remember? Why these? |
| **Environmental context and resources (8)** |
| - Are there any aspects of your clinic environment that affect your ability to… (Prompt: physical infrastructure, financial, human, resource factors)  1. Assess people’s level of risk for sedentary behaviour/physical inactivity [with risk screening tools]? 2. Provide tailored SLAMM advice to patients? |
| - How do competing tasks or time constraints affect your ability to…  1. Assess people’s level of risk for sedentary behaviour/physical inactivity [with risk screening tools]? 2. Provide tailored SLAMM advice to patients? |
| **Social influences (9)** |
| - You work alongside other GPs, practice nurses, allied health staff etc. - How do they influence whether or not you proactively address patients sedentary/physical activity behaviours [prompt for assess/provide tailored SLAMM interventions]? |
| - Have you observed anyone doing this well? |
| - Does your workplace support you to proactively assess and provide tailored advice regarding sedentary behaviour/physical inactivity to your patients? |
| **Emotion (10)** |
| - Do your emotions/mood (positive or negative) ever influence you to assess and provide tailored advice regarding sedentary behaviour/physical inactivity? Explain. (Examples of emotions: contentment, stress, fatigue, overwhelmed, optimistic). |
| - Does the thought of not addressing sedentary behaviour/physical inactivity create any feelings of worry or concern in you? |
| **Reinforcement (11)** |
| - Are there any incentives/rewards that influence the proactive assessment of sedentary behaviour/physical inactivity? - Are there any incentives/rewards that influence the provision of tailored SLAMM advice/care? |
| - If not: what incentives/rewards would enhance the assessment and provision of tailored advice regarding sedentary behaviour/physical inactivity? |
| **Intentions (12)** |
| - In the next month, how strong is your intention to proactively assess and provide tailored SLAMM advice? (Prompt for further explanation) |
| **Optimism (13)** |
| - We’ve talked about some of the difficulties in assessing and providing tailored advice to patients regarding SLAMM - how optimistic are you that assessing and providing tailored advice according to the SLAMM recommendations would improve outcomes for patients? |
| **Behavioural regulation (14)** |
| - Are there any procedures/steps or ways of working that would encourage you and your colleagues to proactively assess and provide tailored SLAMM advice? (Prompt for planning, systems approaches, reminders) |

- Do you have anything else you would like to add about what we have discussed today?
- Would you like to receive a summary of the findings on completion?

Thank you very much for your time.
